# Deep transcriptome-sequencing and proteome analysis of the hydrothermal vent annelid *Alvinella pompejana* identifies the CvP-bias as a robust measure of eukaryotic thermostability

**DOI:** 10.1186/1745-6150-8-2

**Published:** 2013-01-16

**Authors:** Thomas Holder, Claire Basquin, Judith Ebert, Nadine Randel, Didier Jollivet, Elena Conti, Gáspár Jékely, Fulvia Bono

**Affiliations:** 1 Max-Planck-Institute for Developmental Biology, Spemannstr. 35, Tübingen, D-72076, Germany; 2 Equipe GAME, UMR 7144 CNRS-UPMC, Station Biologique, Place Georges Teissier, Roscoff, 29680, France; 3 Max-Planck-Institute of Biochemistry, Department of Structural Cell Biology, Am Klopferspitz 18, Martinsried, D-82152, Germany

## Abstract

**Background:**

*Alvinella pompejana* is an annelid worm that inhabits deep-sea hydrothermal vent sites in the Pacific Ocean. Living at a depth of approximately 2500 meters, these worms experience extreme environmental conditions, including high temperature and pressure as well as high levels of sulfide and heavy metals. *A. pompejana* is one of the most thermotolerant metazoans, making this animal a subject of great interest for studies of eukaryotic thermoadaptation.

**Results:**

In order to complement existing EST resources we performed deep sequencing of the *A. pompejana* transcriptome. We identified several thousand novel protein-coding transcripts, nearly doubling the sequence data for this annelid. We then performed an extensive survey of previously established prokaryotic thermoadaptation measures to search for global signals of thermoadaptation in *A. pompejana* in comparison with mesophilic eukaryotes. In an orthologous set of 457 proteins, we found that the best indicator of thermoadaptation was the difference in frequency of charged versus polar residues (CvP-bias), which was highest in *A. pompejana*. CvP-bias robustly distinguished prokaryotic thermophiles from prokaryotic mesophiles, as well as the thermophilic fungus *Chaetomium thermophilum* from mesophilic eukaryotes. Experimental values for thermophilic proteins supported higher CvP-bias as a measure of thermal stability when compared to their mesophilic orthologs. Proteome-wide mean CvP-bias also correlated with the body temperatures of homeothermic birds and mammals.

**Conclusions:**

Our work extends the transcriptome resources for *A. pompejana* and identifies the CvP-bias as a robust and widely applicable measure of eukaryotic thermoadaptation.

**Reviewer:**

This article was reviewed by Sándor Pongor, L. Aravind and Anthony M. Poole.

## Background

*Alvinella pompejana* is one of the most heat tolerant of all animals known to date [1]. This annelid worm inhabits deep-sea hydrothermal vent chimney walls in self-made glycoprotein tubes [2], where it is exposed to extreme environmental conditions (high pressure, high temperature, low pH, anoxia, heavy metals). *In situ* measurements inside occupied tubes, near the animals’ tails, revealed temperatures of approximately 68°C, compared to temperatures of approximately 20°C in the surrounding water [3]. Given the steep temperature gradient inside the tubes and the difficulty of carrying out such *in situ* measurements, the maximum body temperature *A. pompejana* can tolerate is unclear [1]. Direct temperature preference and tolerance experiments using a high-pressure aquarium with a thermal gradient have not yet been carried out on adult *A. pompejana*. However, such experiments have shown that a North-Pacific relative of *A. pompejana*, *Paralvinella sulfincola,* prefers temperatures between 40°C and 50°C and tolerates temperatures up to 55°C [4]. The habitat of *A. pompejana* is similar to that of *P. sulfincola*, and it is likely that adult *A. pompejana* have a similar thermal preference. Given its high temperature tolerance, there has been considerable interest in studying the mechanisms of thermoadaptation in *A. pompejana*, and in establishing sequence-resources for this organism [5].

A thermoadapted metazoan proteome could greatly benefit structural biology research. The advantage of using thermostable proteins for structural studies has been well documented. In general, proteins from thermophiles are more stable and less flexible than their mesophilic counterparts. Consequently, these proteins are more amenable for expression, purification and crystallization experiments, and have often been used to solve the structure of large macromolecular complexes. Well-known examples include the ribosome, whose complete atomic structure was first determined from the thermophilic eubacterium *Thermus thermophilus*[6], and the exosome, first purified and crystallized from the archaebacterium *Sulfolobus solfataricus*[7]. Among the eukaryotes, the fungus *Chaetomium thermophilum* has recently been shown to have a thermoadapted proteome, and this facilitated the structural study of nuclear pore components [8]. Among the metazoans, *A. pompejana* potentially represents a promising source of thermostable proteins and complexes.

Although *A. pompejana* was first described more than 30 years ago, biochemical data have only been published for a small subset of its proteins. Melting temperature (Tm) values, the most direct measure of thermal stability, have only been published for cuticular and interstitial collagen [9,10]. These proteins have melting temperatures of 45–46°C, 17 degrees higher than that of collagens from shallow seawater annelids [9,10]. The activity of *A. pompejana* and human recombinant DNA polymerase η (Pol η) following high temperature incubation has also been tested. *A. pompejana* Pol η maintained high activity following incubation at temperatures up to 49°C, whereas human Pol η maintained high activity only until 43°C. At 52°C the activity of Pol η from both species dropped below 20% [11]. *A. pompejana* superoxide dismutase (SOD) was also shown to have enhanced chemical stability relative to its human counterpart by guanidine denaturation, a measure thought to correlate with thermal stability. In this study the structure of *A. pompejana* SOD was also solved at high resolution and for the first time in complex with H_2_O_2_[12]. The *A. pompejana* splicing factor U2AF65 in complex with RNA has also been shown to have a slightly increased thermal stability (using RNA-binding as a readout) relative to the human protein (6°C higher) [13].

The aforementioned studies showed increased thermostability of some *A. pompejana* proteins. In contrast, other studies either did not reveal higher thermal stability for *A. pompejana* proteins [14], or measured parameters (e.g. optimal temperatures for enzyme activity) that only allowed an indirect assessment of thermal stability. For example, the extracellular giant hemoglobin of *A. pompejana* showed higher oxygen affinity than that of other annelids, and exhibited other functional properties related to the vent environment [15], but its macromolecular assembly was unstable at 50°C, and it was not more thermostable than earthworm hemoglobin [14].

Given the small number of biochemical studies and the uncertainties about the thermotolerance of the animals, the general degree of thermoadaptation of the *A. pompejana* proteome is still unclear. Sequence analysis of a large number of proteins could reveal general features of thermoadaptation. There have been many attempts to correlate protein thermal stability with sequence or structure derived features [16]. When comparing sequence composition of thermophiles and mesophiles, the most apparent difference is an enrichment of charged residues in combination with a decrease in the number of polar residues in the thermophilic proteins. Both the (E + K)/(Q + H) ratio and the CvP-bias (difference in the frequency between charged and polar residues) can discriminate hyperthermophiles from mesophiles [17] as well as barophiles [18]. Another study identified a universal set of residues, I,V,Y,W,R,E,L (IVYWREL measure), enriched in thermophiles [19]. More complex discrimination functions have also been proposed, such as the function employed by THERMORANK [20]. This method uses a linear combination of 10 sequence-based features to rank a set of input sequences by relative thermostability. Another method, the Tm-Index tool, uses dipeptide composition to predict a melting point index for a single protein sequence [21]. These measures have not yet been systematically tested on the *Alvinella* proteome.

Recently, a large *A. pompejana* cDNA resource, prepared from three different tissues and whole animals was published [5]. Analysis of this dataset in comparison with various metazoan homologs showed that *A. pompejana* protein sequences have the highest proportion of charged amino acids. This bias was interpreted as a sign of protein thermostability [5]. Another EST resource, generated by the Joint Genome Institute (JGI, http://www.jgi.doe.gov/), is also publicly available. These *A. pompejana* EST resources could form the basis for reconstituting and determining structures of metazoan proteins and multiprotein complexes. However, in order for structural biologists to identify all components of large multiprotein complexes, where the lack of a single component will impede reconstitution, more extensive sequence coverage is essential.

To increase the available dataset, we first carried out deep sequencing of the *A. pompejana* transcriptome. Using this resource and the published sequences, we established a large orthologous dataset with a taxon sampling that included other annelids, as well as the thermostable fungus, *Chaetomium thermophilum*[8]. To investigate the extent of thermoadaptation of *A. pompejana* we then performed a systematic survey of the available sequence-based thermostability measures previously established for prokaryotes on its proteome. Testing the THERMORANK, IVYWREL, Tm-Index, (E + K)/(Q + H), and loop-length gave conflicting results. Our analyses identified the CvP-bias as the best measure to discriminate *A. pompejana* from mesophilic eukaryotes. The CvP-bias also discriminated the thermophilic fungus *C. thermophilum* from mesophilic eukaryotes. The correlation of the CvP-bias with thermostability was also supported by experimentally-determined thermostability data.

## Results

### Deep sequencing of the *A. pompejana* transcriptome

To gain insights into the mechanisms of thermoadaptation of *A. pompejana,* we first performed deep sequencing of its transcriptome. We used a combination of the Sanger, Roche/454 and Illumina technologies. With the 454 technology we obtained 2,717,445 reads of an average length of 220 bp. Using Illumina paired-end sequencing (~300 bp fragments) we obtained 87 million reads of an average length of 76 bp. These resources, and an additional 10,063 novel Sanger ESTs we generated, were assembled into a reference transcriptome dataset. Large-scale EST sequencing projects have also been carried out by the Joint Genome Institute (JGI) and Genoscope [5], yielding a total of 218,458 publicly available ESTs (as of Feb 2011). We also assembled these sequences with our resource, creating a combined MPI + JGI + Genoscope dataset.

**Figure 1:**
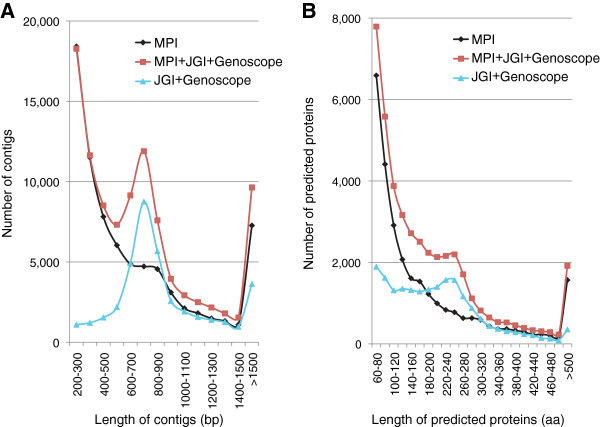
**Size distribution of cDNA contigs and predicted protein sequences in three *A. pompejana *datasets.** (**A**) Histograms representing the number of contigs in the different size-ranges for the MPI (black), MPI + JGI + Genoscope (red) and JGI + Genoscope (cyan) assemblies. (**B**) Histograms representing the number of predicted proteins in the different size-ranges for the MPI (black), MPI + JGI + Genoscope (red) and JGI + Genoscope (cyan) datasets.

To estimate the number of novel sequences in our resource (MPI dataset, Additional file 1) we compared it with the already available ESTs (JGI + Genoscope dataset, Additional file 2), as well as the combined dataset (MPI + JGI + Genoscope dataset, Additional file 3). First, we compared the length distribution of contigs and singletons in the three assemblies (Figure 1A). The final combined assembly nearly doubled the available transcriptome resources for *A. pompejana*, with 60,475 contigs longer than 500 bp, compared to 34,860 in the JGI + Genoscope dataset. Due to the small size of the 454 and Illumina reads, the MPI and the MPI + JGI + Genoscope datasets were dominated by short contigs (Figure 1A). However, they also contained a larger number of long contigs (>1,500 bp) than the JGI + Genoscope dataset (7,269 in MPI and 9,639 in MPI + JGI + Genoscope compared to 3,634 in JGI + Genoscope).

### Annotation of predicted *A. pompejana* proteins

Next we searched the nucleotide datasets for potential open reading frames and analyzed the predicted protein sequences (Additional file 4,  5 and 6). We identified many novel full-length and partial sequences (Figure 1B and Table 1), and also extended the length of already known partial proteins.

**Table 1:** **Size of *A. pompejana *protein datasets**

**Dataset**	**% identity**	**#full-length**	**#partial with stop**	**#total**
MPI (New data)	100	6 272	15 886	28 169
	98	5 778	14 893	26 992
	90	5 667	14 502	26 433
JGI + Genoscope (Existing data)	100	6 233	15 539	23 962
	98	5 360	13 365	19 890
	90	5 008	12 341	18 155
MPI + JGI + Genoscope (Combined data)	100	10 778	26 068	42 665
	98	9 359	23 131	38 185
	90	8 722	21 288	35 235

Number of full-length (with start and stop codon), partial (with stop codon), and total number of predicted protein sequences in the three datasets clustered at 100%, 98% and 90% identity.

We then compared the *A. pompejana* predicted proteins in the MPI + JGI + Genoscope dataset to those already available in the JGI + Genoscope dataset. We found 13,301 sequences (>100 aa) in the MPI + JGI + Genoscope dataset with no identical BLAST hit (fraction identical <90%) to the JGI + Genoscope dataset. We then performed BLASTP searches with these sequences in the predicted proteome of the annelid *Capitella teleta,* the closest relatives of *A. pompejana* with complete genome information (phylogenetic tree in Additional file 7). We found that 3,897 *C. teleta* sequences had one or more significant hits (e-value 1e-5) among the novel *A. pompejana* proteins. These represent newly identified, conserved *A. pompejana* proteins.

In addition, our data also extended the length of many truncated protein sequences in the JGI + Genoscope dataset. In a BLASTP comparison of the predicted protein datasets, 2,776 query sequences from the MPI + JGI + Genoscope dataset were at least 40 amino acids longer than their corresponding fragments in the JGI + Genoscope dataset.

Next, we annotated the combined predicted *A. pompejana* proteome using BLASTP (Additional file 8). We defined the sequences based on their first hit in the SwissProt database, and further annotated them with the first hit in the *C. teleta*, human, *Danio rerio* and *Drosophila melanogaster* proteomes. We also identified 775 sequences with no BLASTP hit in 26 eukaryotic genomes (including 18 animals) but significant hits in prokaryotes (in the UniRef90 database). These were annotated as potential contaminants or genes that originated by recent lateral gene transfer. A future *A. pompejana* genome sequencing project could distinguish between these two possibilities.

In order to estimate the completion of the *A. pompejana* datasets, we performed extensive BLASTP searches in the proteome of 15 animal and 8 additional eukaryotic species. We used *C. teleta* protein sequences as query and performed BLASTP searches in the 23 eukaryotic datasets, including the *A. pompejana* datasets. We then counted the number of *C. teleta* proteins that had a significant hit (e-value 1e-5) in all animals, but not outside animals, or all eukaryotes. These two subsets of proteins (general animal and general eukaryotic) are also expected to be present in *A. pompejana*. We therefore counted how many of these two subsets had a BLAST hit in the *A. pompejana* datasets (Table 2). We found that the combined *A. pompejana* resource is about 74-99% complete, depending on the subset of proteins examined. Animal-specific proteins were highly covered, between 74-94%, depending on whether we considered all sequences or only full-length sequences. Proteins present in all eukaryotes had even higher percent coverage (87-99%). This is probably due to the higher expression levels of genes with general eukaryotic cellular functions. When we considered only full-length *A. pompejana* proteins, the MPI + JGI + Genoscope dataset was 74% complete for animal-specific proteins, compared to the 55% completion of the JGI + Genoscope dataset. These searches show that the *A. pompejana* resource is about 74-99% complete at the level of eukaryotic paralogous families and protein domains. Overall, our sequencing efforts greatly extended the known *A. pompejana* transcriptome and proteome.

**Table 2:** **Estimated completion of the *A. pompejana *proteome**

	***C. teleta***	***A. pompejana***					
		**JGI + Genoscope (Existing data)**	**MPI (New data)**	**MPI + JGI + Genoscope (Combined data)**	**JGI + Genoscope full length (Existing data)**	**MPI full length (New data)**	**MPI + JGI + Genoscope full length (Combined data)**
Hits to all 14 animals	1164 (100%)	932 (80%)	1017 (87%)	1074 (92%)	639 (55%)	708 (61%)	859 (74%)
Hits to all 22 eukaryotes	3958 (100%)	3862 (98%)	3849 (97%)	3922 (99%)	3036 (77%)	3179 (80%)	3454 (87%)

Number of BLAST hits in the different *A. pompejana* datasets using two subsets of the *Capitella* proteome: sequences that only give hits in all of the 14 animals included, but not outside metazoa (hits to all 14 animals only), and sequences that have hits in all 14 animals included and 8 other non-metazoan eukaryotes. Total number of hits is given as well as the percentage of a hit relative to the total number of *Capitella* queries (100%).

### Generation of an orthologous set of protein sequences

As a prerequisite for a thorough assessment of the thermoadaptation of the *A. pompejana* proteome we generated a large set of orthologous protein sequences from *A. pompejana* and nine other eukaryotic species. Importantly, our orthologous set also included three other lophotrochozoan species, the annelids *C. teleta* and *Helobdella robusta* and the mollusk *Lottia gigantea*. To be able to identify general features of thermoadaptation in eukaryotes, we also included the thermotolerant fungus, *Chaetomium thermophilum*, and a mesophilic yeast, *Saccharomyces cerevisiae* in the orthologous set.

The orthologous set was generated using an all-against-all BLASTP search and the reciprocal best-hit approach, and contained 457 members (Additional file 9). We used this dataset to search for signals of thermoadaptation using a broad range of sequence-based methods that have been proposed in the thermostability literature.

### Sequence based thermostability ranking

Protein sequence composition of thermophilic prokaryotes differs significantly from mesophilic protein sequences. The general trend of an increased number of charged residues and a decreased number of polar residues was reported several times [16,17,22].

It was previously reported that *A. pompejana* proteins were enriched in charged amino acids [5]. This was interpreted as a sign for enhanced thermostability. Our set of orthologous sequences only partly supports this observation. Compared to the protein sequences from *Drosophila melanogaster*, *Danio rerio* and *Homo sapiens*, *A. pompejana* is strongly enriched in Lys, and slightly enriched in Asp, but not in Glu or Arg. However, the enrichment in Lys and Asp is shared with *H. robusta* and *L. gigantea*, two other lophotrochozoan species that do not live at high temperatures. These two species also have fewer Ala than *A. pompejana*, a sequence feature that has previously been proposed to be associated with thermostability [20].

**Figure 2:**
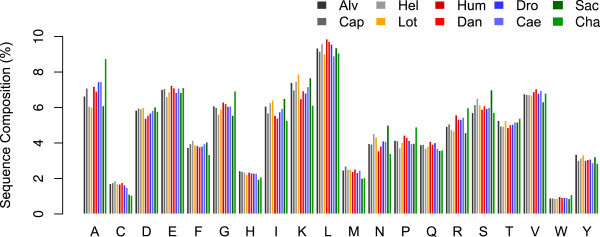
**Amino acid compositions in the orthologous protein datasets of the analysed species.** Percent of amino acid content calculated from the orthologous set of 457 protein sequences in the 10 species: *Alvinella pompejana* (Apo)*, Capitella teleta* (Cap)*, Helobdella robusta* (Hel), *Lottia gigantea* (Lot)*, Homo sapiens* (Hum), *Drosophila melanogaster* (Dro), *Danio rerio* (Dan), *Caenorhabditis elegans* (Cae), *Saccharomyces cerevisiae* (Sac), *Chaetomium thermophilum* (Cha).

The fungus *C. thermophilum* is strongly enriched in Ala, Gly, Pro, and Arg. This reflects the high GC-content of this species (see below). However, the enrichment in Arg is compensated by a reduction in Lys content, resulting in no global enrichment in charged residues in *C. thermophilum*. We conclude that the number of charged residues alone does not distinguish *A. pompejana* and *C. thermophilum* from mesophilic species (Figure 2).

**Figure 3:**
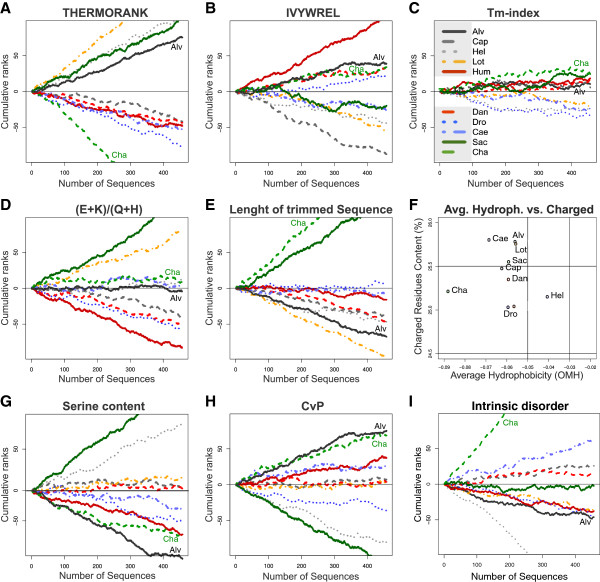
**Cumulative ranking of the orthologous protein set from the analyzed species based on various thermostability measures.** (**A-E**) Cumulative ranks for the orthologus set based on (**A**) THERMORANK, (**B**) IVYWREL, (**C**) Tm-index, (**D**) (E + K)/(Q + H), (**E**) length of trimmed sequence in alignment. (**F**) Average hydrophobicity versus charged residue content for the orthologous set. (**G-I**) Cumulative ranks for the orthologus set based on (**G**) Serine content, (**H**) CvP-bias, and (**I**) intrinsic disorder (IUPred score [25]). The species are as in Figure 2.

We next performed sequence-based thermostability ranking calculations using a variety of other computational methods. All measures were used to rank the whole orthologous set of 457 proteins. We found that THERMORANK [20], “Tm Predictor” [21], the IVYWREL measure [19] and the (E + K)/(Q + H) ratio [23], all failed to rank *A. pompejana* or *C. thermophilum* as the most thermostable species (Figure 3A-D). In the THERMORANK analysis, for example, *A. pompejana* ranks behind *H. robusta* and *L. gigantea*, also highlighting the importance of broad taxon-sampling when performing these comparisons. Additionally, the length of the protein sequences in the trimmed multiple alignments (an indication of the length of surface loops) [24] did not identify *A. pompejana* proteins as thermostable, and ranked the two fungi as the species with the most compact proteins (Figure 3E). The average hydrophobicity versus charged residues, a measure that showed *A. pompejana* as an outlier [5], clustered *A. pompejana* with mesophilic *L. gigantea* (Figure 3F). A reduction in intrinsic protein disorder may also correlate with thermoadaptation. However, when we performed protein disorder predictions [25] on the orthologous set, *A. pompejana* ranked as average. *C. thermophilum* ranked highest (Figure 3I), probably due to the high content of Pro, Ala, Gly and Arg, residues that strongly contribute to structural disorder [25]. 

We found only two thermostability measures that weakly discriminated *A. pompejana* and *C. thermophilum* from the other species. Low serine content [26] ranks *A. pompejana* and *C. thermophilum* first and second in cumulative rankings, although *C. thermophilum* receives very similar scores to human (Figure 3G). The CvP-bias [17] was the only measure that ranked *A. pompejana* and *C. thermophilum* as the two most thermostable species (Figure 3H).

**Figure 4:**
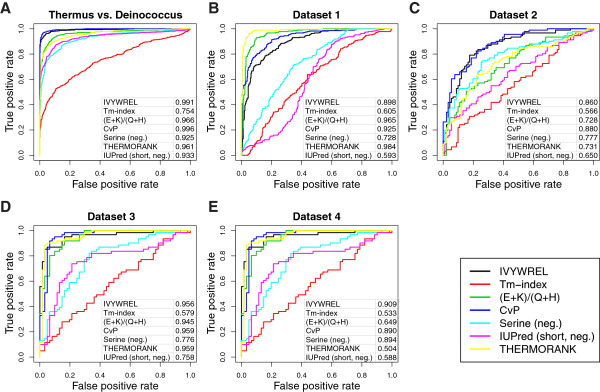
**Thermostability measures in prokaryotic thermophile-mesophile ortologous protein datasets.** (**A-E**) Receiver operating characteristic (ROC) curves for (hyper-)thermophile-mesophile homologous protein pairs from (**A**) one self-compiled dataset (*Thermus thermophilus* vs. *Deinococcus radiodurans*) and (**B-E**) four published datasets were tested with the different thermostability measures to discriminate thermophiles from mesophiles. Dataset 1 (**B**) is from ref. [20], dataset 2 (**C**) is from ref. [27], dataset 3 and 4 (**D, E**) are from ref. [28] (containing hyperthermophiles versus mesophiles and thermophiles versus mesophiles respectively).

We validated all the measures used on prokaryotic thermophile-mesophile test datasets. All measures robustly discriminated a self-compiled *Thermus thermophilus* (a thermophilic bacterium) vs. *Deinococcus radiodurans* (an extremophilic but not thermophilic bacterium) dataset. Other published datasets were also tested, and most measures distinguished thermophiles from mesophiles [20,27,28] (Figure 4). 

**Figure 5:**
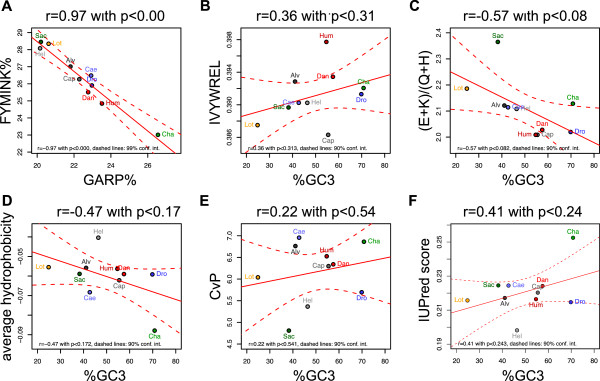
**Influence of GC-content on amino acid composition and thermostability measures.** (**A**) Plot of the frequency of GC-rich codons (GARP) against the frequency of AT-rich codons (FYMINK) in the species of the orthologous set. (**B**) Average IVYWREL, (**C**) (E + K)/(Q + H), (**D**) average hydrophobicity, (**E**) CvP-bias, (**F**) and IUPred values plotted as a function of GC3-content (GC-content at the third codon position) in the species of the orthologous set. The regression lines with 95% confidence intervals and correlation coefficients with p-values are shown.

We also calculated the GC-content for the nucleotide sequences corresponding to the protein sequences in our trimmed orthologous set (Table 3). We found that *C. thermophilum* had the highest GC-content, *A. pompejana* had a GC-content below the average of the dataset, and *L. gigantea*, *H. robusta* and *S. cerevisiae* had the lowest GC-content values. We plotted the ratio of GC-rich codons (GARP residues) against the ratio of AT-rich codons (FYMINK residues; Figure 5A) [29] across the ortologous set and found a very strong correlation, indicating that GC-content strongly influences amino acid composition. We also tested how individual amino acids are influenced by GC-content and found that the frequency of GARP residues as well as of Trp, Ile and Lys correlates with GC-content (Additional file 10). The thermostability measures we applied, however, do not correlate with GC-content (Figure 5B-F), indicating that the observed ranking is not influenced by GC-content. 

**Table 3:** GC-content of nucleotide sequences corresponding to the trimmed orthologous protein set

**Species**	**GC (%)**			**GC enrichment relative to mean**			**GC total (%)**
	**#1**	**#2**	**#3**	**#1**	**#2**	**#3**	
*H. sapiens*	56.23	38.06	54.67	1.05	1.02	1.09	49.65
*S. cerevisiae*	46.54	35.51	38.25	0.87	0.95	0.76	40.1
*C. elegans*	54.83	37.63	42.69	1.03	1.01	0.85	45.05
*C. teleta*	54.46	37.32	55.46	1.02	1	1.11	49.08
*H. robusta*	49.2	35.62	46.4	0.92	0.96	0.93	43.74
*D. melanogaster*	56.98	37.57	69.85	1.07	1.01	1.39	54.8
*A. pompejana*	52.2	36.42	41.08	0.98	0.98	0.82	43.23
*C. thermophilum*	59.75	41.02	70.83	1.12	1.1	1.41	57.2
*D. rerio*	55.62	37.68	57.52	1.04	1.01	1.15	50.27
*L. gigantea*	47.59	35.77	24.82	0.89	0.96	0.49	36.06
mean	53.34	37.26	50.16				46.919

GC-content (total and at different codon positions) in the orthologous set and GC-content enrichment, relative to the mean of each species studied at each position.

### Close correlation of the CvP value with experimentally determined thermal stabilities

Out of all the sequence-based thermostability measures used, only the CvP-bias ranked both *A. pompejana* and *C. thermophilum* highest. To test how well this measure correlates with thermal stability, we calculated the CvP-bias for proteins with experimentally determined protein stabilities (Table 4). Despite limited sample size, the CvP-bias values correlated well with experimentally determined stabilities for *A. pompejana* Pol η [11], collagen [9,10] and U2AF65 [13], and for *C. thermophilum* Nup170, Nup192 [8], and xylanase [30], when compared to mesophilic proteins. 

**Table 4:** Correlation of CvP-bias differences with experimentally determined Tm value differences for various orthologous protein pairs

**Protein**		**Thermophilic (*A. pompejana *or *C. thermophilum*)**	**Mesophilic (human,*S. cerevisiae*or*T. reesei*)**	**Difference**
Rrp4 (*A. pompejana* vs. human)	CvP-bias	0.408	6.122	5.714
	Tm	38°C	43.1°C	5.1
Pol η (*A. pompejana* vs. human)	CvP-bias	5.71	3.67	−2.04
	Tm	49°C	43°C	−6
Collagen (*A. pompejana* vs. human)	CvP-bias	8.696	5.804	−2.892
	Tm	45-46°C	38-42°C	−3 to −8
U2AF65 (*A. pompejana* vs. human)	CvP-bias	11.90	5.68	−6.22
	Tm	47°C	43°C	−4
Nup170 (C*. thermophilum* vs. *S. cerevisiae*)	CvP-bias	−1.13	−4.86	−3.73
	Tm	57°C	36°C	−21
Nup192 (C*. thermophilum* vs. *S. cerevisiae*)	CvP-bias	3.47	−3.33	−6.8
	Tm	57°C	36°C	−21
Xylanase (C*. thermophilum* vs. *Trichoderma reesei*)	CvP-bias	−20.276	−22.523	−2.24
	Tm	60°C	50°C	−10

Validation of CvP-bias with experimentally determined thermal stabilities for orthologous protein pairs from refs. [8,30].

**Figure 6:**
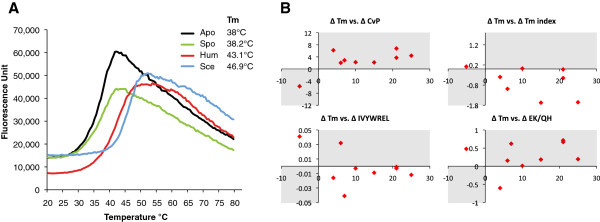
**Experimental validation of the CvP-bias as a predictor of thermal stability.** (**A**) Thermal denaturation curves for *A. pompejana* (Apo), human (Hum), *Saccharomyces cerevisiae* (Sce) and *Schisosaccharomyces pombe* (Spo) recombinant Rrp4 proteins. (**B**) Correlation between CvP-bias difference and Tm difference in orthologous protein pairs with experimentally determined Tm values from *A. pompejana* and *C. thermophilum*. Shading indicates the areas where the values are expected, if the predictors work. Related to Table 4.

To further test the reliability of the CvP-bias measure, we searched for *A. pompejana* proteins with the lowest CvP-bias ranking among metazoans. We identified 26 proteins where *A. pompejana* ranked lowest, including the exosome component Rrp4. We expressed and purified recombinant Rrp4 proteins from *A. pompejana,* human, and yeast and performed thermal denaturation experiments. As predicted, we found that human Rrp4 was more thermoresistant than the *A. pompejana* protein (Figure 6A and Table 4).

Overall, the difference in the thermal stability values between the thermophilic and mesophilic orthologous pairs and the difference in the CvP-bias showed a consistent trend. The proteins that showed higher thermal stability than their ortholog consistently showed a higher CvP-bias (Figure 6B and Table 4). Three other measures (Tm-Index, IVYWREL, and (E + K)/(Q + H)) did not show such a trend. These results suggest that the CvP-bias, in comparison to mesophilic orthologs, is a good predictor of the thermal stability of eukaryotic proteins.

### Close correlation of the CvP value with the body temperatures of homeothermic vertebrates

The thermal stability experiments performed to date indicate that *A. pompejana* proteins have approximately 4–8°C higher thermal denaturation values than their mesophilic counterparts. Nevertheless the CvP-bias was able to discriminate *A. pompejana* from mesophilic animals, indicating that it is a sensitive measure of thermoadaptation. Different homeothermic vertebrates can also show up to 5°C difference in body temperature, with dolphins and whales having a body temperature of 36°C and birds 41°C. To test whether the CvP-bias also correlated with body temperatures among the homeothermic vertebrates we analysed the mean CvP-bias of the proteomes of 10 mammalian and bird species. We found a significant correlation between body temperature of a species and the mean CvP-bias of its proteins (Pearson’s r = 0.71, p = 0.02). In contrast, the Tm-Index, IVYWREL, and intrinsic disorder did not show significant correlation (Table 5).

**Table 5:** Correlation of CvP-bias with the body temperature of homeothermic vertebrates

**Species**	**Number of sequences**	**CvP-bias**	**Tm-Index**	**IUPred**	**IVYWREL**	**Body temperature °C**
HUMAN	50 006	0.93	0.9	0.31	36.87	37°C
MOUSE	36 368	0.97	0.93	0.29	37.16	37°C
TURTR (bottle-nosed dolphin)	307	0.99	0.79	0.28	38.8	36.8°C
RAT	30 926	1.02	0.97	0.28	37.17	37°C
BOVIN	23 358	1.34	0.96	0.27	37.6	37°C
CHICK	19 671	1.42	0.94	0.28	37.3	41°C
CANFA (dog)	24 002	1.43	0.96	0.28	37.5	37°C
PIG	24 692	1.61	0.98	0.28	37.43	37°C
MELGA (turkey)	15 899	1.72	0.94	0.27	37.66	41°C
TAEGU (zebra finch)	16 717	2.32	0.97	0.27	37.72	41°C
	**Pearson’s r**	0.71	0.23	−0.44	0.02	

Validation of CvP-bias with body temperatures of homeothermic vertebrates. The number of sequences as well as the mean values of the indicated measures are shown for 10 vertebrate species. Pearson’s correlation coefficients of thermostability measures to body temperature are shown.

## Discussion

Taking advantage of an extended sequence dataset, we searched for global signals of thermoadaptation in the proteome of the hydrothermal vent annelid *A. pompejana*. We used a broad phylogenetic framework, comparing orthologous eukaryotic proteins across vast evolutionary distances. For a detailed understanding of thermoadaptation, one would ideally focus on several closely related thermophilic and mesophilic species. However, there are no similar large-scale sequence resources available from other alvinellids, and such analyses are also confounded by the uncertain evolutionary history of thermoadaptation within the group. A recent analysis compared *A. pompejana* to a closely related mesophilic species, *Paralvinella grasslei*[29]. This study revealed a higher proportion of Ala residues in *A. pompejana* than in *P. grasslei*. Given the possibility that *P. grasslei* may only recently have become a mesophile, evolving from a thermophilic common ancestor with *A. pompejana*, the direction of change and the role of Ala in thermoadaptation in alvinellids are unclear. Only an increased taxon sampling and further analyses within and outside the alvinellids will clarify this. In our orthologous set the proportion of Ala residues in *A. pompejana* is not higher than in most metazoans. The thermophilic *C. thermophilum* is highly enriched in Ala, but this is probably due to the high GC-content of this species.

Our approach compared *A. pompejana* to a broad selection of taxa, including other annelids, as well as very distantly related eukaryotes (fungi). This global comparison indicated that the CvP-bias may be a robust measure of thermostability across eukaryotes. CvP-bias ranked both *A. pompejana* and the thermophilic fungus *C. thermophilum* with the highest score among 10 eukaryotic species. Importantly, these ranking results cannot be explained by differences in GC-content, given that GC-content and CvP-bias are not correlated.

We also tested the CvP-bias against the limited biochemical data available, and found a good correlation between thermal stability and CvP-bias, when comparing orthologous protein pairs. For a thorough validation or for the development of other, more sensitive measures, more biochemical and genomic data will be needed.

One surprising finding was that the CvP-bias could also predict whether an *A. pompejana* protein would be less thermostable than its human ortholog, as shown for Rrp4. This suggests that not all *A. pompejana* protein are thermoadapted. The thermoadaptation of a subset of proteins with certain functions (e.g. collagen, a major component of the cuticle), together with the up-regulation of heat-shock proteins [5], may be sufficient to enable *A. pompejana* to cope with higher temperatures.

## Conclusions

Our deep-sequencing efforts greatly enhance the existing transcriptome data for *A. pompejana*, nearly doubling the number of full-length cDNAs. This extended resource will be valuable for further comparative genomic studies of metazoans and extremophiles. The correlation of the CvP-bias with thermal stability may be used to identify the most thermoadapted proteins from *A. pompejana* and other thermophilic eukaryotes, potentially facilitating protein structure determination studies.

## Methods

### Alvinella pompejana samples

Samples were collected from the hydrothermal vent chimney sites Julie and Parigo at 13N°/EPR (East Pacific Rise) during the cruises HOT 1996 and PHARE 2002 in the Pacific Ocean using the telemanipulated arms of the submersible Nautile and the ROV Victor. The samples were brought back to the surface into an insulated basket and snap-frozen in liquid nitrogen and stored at −80°C until RNA extraction.

### *A. pompejana* transcriptome sequencing, assembly and analysis

For the sequencing of the *A. pompejana* transcriptome we used a combination of techniques. We generated a custom, normalized, full-length cDNA library (with m^7^Gppp affinity purification to limit bacterial RNA contamination; Invitrogen), cloned into the pENTR222.1 vector from two adult worms. After plating, we sequenced 10,063 randomly picked clones using the Sanger technology (ABI 3730) and the M13-FP primer. The programs Phred and Cross-match were used for base calling and vector trimming.

We also performed 454 sequencing (GS FLX, Roche/454) on the PCR-amplified cDNA library, following concatenation and fragmentation. After adaptor trimming (pDONR), quality (0.05), and length filtering (50 bp cutoff) with the software package CLC Genomics Workbench 4.5.1, we obtained 2,717,445 reads of an average length of 220 bp.

For Illumina sequencing we used total RNA isolated from a third animal, a frozen *A. pompejana* male using the RNaesy Kit (Qiagen), following the breaking up of the tissue (pieces of 20–30 mg). We performed paired-end sequencing following reverse transcription of total RNA using the Smart cDNA Construction Kit (Clontech), m^7^cap-primed second strand synthesis, co-ligation and nebulization of cDNA, gel fractionation (approximately 350 bp) and adapter ligation. Samples were run on a HiSeq 2000 sequencer obtaining 91,670,518 paired reads. After adaptor trimming (pDONR222, Illumina PCR Primer, Illumina Paired End PCR Primer 2.0), quality (0.05) and length (30 bp cutoff) filtering, using the CLC Genomics Workbench 4.5.1, we obtained 87,799,426 high-quality reads of an average length of 76 bp.

All quality trimmed and vector screened 454 and Illumina reads were assembled using Velvet version 1.1. and Oases [31]. The k-mer length used was 25. The resulting contigs and singletons were joined with all published *A. pompejana* ESTs from NCBI dbEST, and with our EST sequences, and passed to the CAP3 assembler with default parameters [32]. To assess the amount of newly obtained transcripts from our own sequencing effort, our sequencing data and the JGI + Genoscope ESTs (218,458) were also assembled separately with CAP3. Contigs and singletons were joined for each assembly to compose the final set of transcript sequences.

Transcript sequences were translated with ESTScan [33] and trimmed to the longest stop codon-free protein fragment. If a 5’ stop-codon was present, the sequence was scanned to the next methionine and that was considered as the protein start. Fragments shorter than 60 amino acids were discarded. The sequence assemblies can be queried by BLAST at http://jekely-lab.tuebingen.mpg.de/blast/.

Amino acid composition of protein sequences and measures derived from amino acid composition were calculated with custom scripts in Python. The CvP bias was calculated as (C - P)/length × 100, where C and P represents the number of charged (EDKR) and polar residues (STNQ) respectively.

For the multi-species BLAST comparisons *Capitella teleta*, *Helobdella robusta, Lottia gigantea,* and *Daphnia pulex* protein sequences were downloaded from JGI (http://www.jgi.doe.gov/, Filtered Models). Protein sequences for *Caenorhabditis elegans, Drosophila melanogaster, Anopheles gambiae, Strongylocentrotus purpuratus, Branchiostoma floridae, Ciona intestinalis, Danio rerio, Gallus gallus, Homo sapiens, Hydra magnipapillata, Nematostella vectensis, Trichoplax adhaerens, Monosiga brevicollis, Neurospora crassa, Schizosaccharomyces pombe, Dictyostelium discoideum, Naegleria gruberi, Chlamydomonas reinhardtii, Paramecium tetraurelia,* and *Arabidopsis thaliana* were downloaded from the NCBI RefSeq database (http://www.ncbi.nlm.nih.gov/RefSeq/).

### Orthologous sets

We downloaded the Swissprot and TrEMBL sequences for *Homo sapiens*, *Drosophila melanogaster*, *Caenorhabditis elegans*, *Danio rerio*, and *Saccharomyces cerevisiae* from UniprotKB. For *Capitella teleta*, *Helobdella robusta* and *Lottia gigantea* protein sequences were downloaded from JGI (http://www.jgi.doe.gov/, Filtered Models). The predicted *Chaetomium thermophilum* proteins were downloaded from http://ct.bork.embl.de. Each proteome was clustered by 98% sequence identity and arbitrary length difference, using CD-HIT [34]. A total of 45 pairwise BLASTP searches were performed, best hits that were consistent in all searches were considered as an orthologous set, yielding 457 sets. For each orthologous set, a multiple sequence alignment was created using MUSCLE [35]. Because many of the protein sequences were not full length, the alignments were trimmed from both ends to the first column without a gap symbol, so that each protein is represented with the same fragment. The sequences from the trimmed alignments were used for all further analyses (Additional file 9). The tree, based on the recently published phylogeny of annelids [36] and the consensus animal phylogeny [37], in Additional file 7 represents the relationships of the species in the orthologous set.

### Thermophile-mesophile benchmark datasets

Protein sequences for *Thermus thermophilus* and *Deinococcus radiodurans* were downloaded from UniProtKB by filtering on taxonomy ids 262724 and 1299. The Li et al. [20] dataset (Dataset 1, THERMORANK) was downloaded from http://www.abl.ku.edu/thermorank/suppl.html. The Montanucci et al. [27] dataset (Dataset 2) was downloaded from http://lipid.biocomp.unibo.it/~ludovica/thermo-meso-MUT/ and only the first pair was considered from each cluster. The Taylor and Vaisman datasets (Datasets 3 and 4) were obtained from the supplementary material of ref. [28].

### Thermorank analysis

For local usage, the THERMORANK tool was re-implemented according to ref. [20], using the Python programming language. Tripeptide residue accessible surface area values were used as described in ref. [38]. The 8 protein sequences in each orthologous set were ranked and the cumulative rank sums over the 457 sets calculated to assess the overall trend of ranking. Ranking calculations for all measures were performed using the R software environment (http://www.r-project.org/).

### Cloning, expression and purification of *A. pompejana* proteins

*A. pompejana* Rrp4 was cloned and expressed as a His-tagged fusion together with Rrp4 orthologues from *Schisosaccharomyces pombe*, *Homo sapiens* and *Saccharomyces cerevisiae*. DNA was transformed in BL21Gold pLysS (Stratagene), grown overnight at 18°C and induced with 0.5 mM IPTG. Cells were resuspended and lysed by sonication in a buffer containing Tris pH 7.5, 500 mM NaCl, 20 mM Imidazole, 5 mM beta-mercaptoethanol and 10% glycerol. All proteins were purified using affinity chromatography on Talon resin (Clontech) (elution with 250 mM imidazole), followed by size exclusion chromatography on a GF200 column.

### Thermal shift assay

Solutions containing 5 μl of 2 mg/ml protein with 35x of Sypro Orange (Invitrogen) and 45 μl of buffer screen were added to the wells of a 96-well PCR plate (Eppendorf). The plate was sealed and heated in a real-time PCR system (Eppendorf) from 20°C to 80°C in increments of 0.2°C. Fluorescence changes were monitored simultaneously. The wavelengths for excitation and emission were 470 and 550 nm, respectively. To obtain the temperature midpoint for the protein unfolding transition (Tm), a Boltzmann model was used to fit the fluorescence data [39].

### Reviewers’ comments

Reviewer’s reports

Reviewer 1: Sándor Pongor, International Centre for Genetic Engineering and Biotechnology, Trieste, Italy

The authors present transcriptome sequencing studies on the Alvinella pompejana worm that lives in high temperature environments. A. pompejana is an attractive eukaryotic model organism both for studying thermoadaptation and also because thermophylic proteins hold promise for structural studies. The paper presents a significant advance in the sequencing of this organism, a large number of potential ORFs were discovered which warrants publication in itself. The manuscript also presents a variety of data coming from and evaluated by different techniques. The authors also conducted biophysical tests that showed that not all A. pompejana proteins are thermotolerant, indicating that the thermotolerance of this species may not be as outstanding as previously thought.

I recommend the following points to the attention of the authors:

At the first sight, the title and abstract does not make it clear whether or not the CvP index is defined here, or is it already known. I suggest to clarify this, for instance by adding a restrictive adjective, something like “…identifies the CvP-bias as a (reliable, robust) measure of eukaryotic thermostability”. CvP-bias was described in several articles including PMID: 16494505. Since CvP-bias is part of the main message, these papers could be cited in the introduction.

We have now changed the title to “.identifies the CvP-bias as a robust measure…”. We also changed one sentence in the abstract to clearly state that we looked at already known measures “We then performed an extensive survey of previously established prokaryotic thermoadaptation measures” We also included a reference to PMID: 16494505 (ref. 18).

Even though the technical parts are well separated from the main text of the paper, at times the manuscript is still difficult to read, simply because many techniques are used in the project. This could be helped by emphasizing the main messages clarifying the details. For instance, ROC curves presented in Figure 4 could be complemented by a tabular comparison of AUC values. The panels of Figure 4 are too tiny and crowded in my opinion. Also, more details in the Figure legends would improve readability.

We have included the AUC values in Figure 4 and give more details in the figure legends.

Reviwer 2: L. Aravind, National Center for Biotechnology Information, National Library of Medicine, National Institutes 702 of Health, Bethesda, USA

Since its discovery, A. pompejana has been of great interest in regard to the question of how a metazoan might tolerate environmental extremes such as those it faces. Holder et al. use deep transcriptome sequence combined with bioinformatics analysis to attempt to explain the unusual thermotolerance of Alvinella pompejana. A key point made by this study is that the earlier analysis of Alvinella sequences might not have necessarily identified the actual basis for thermostability of proteins in this organism. In particular, the reported features are shared with mesophilic lophotrochozoans, suggesting that they might not be genuine discriminants of thermophily. In this regard, the extended proteome generated from sequence data obtained by the authors is a useful resource. Further, the study shows that the metrics for thermophily that were found to be successful in discriminating prokaryotic thermophily cannot be uncritically applied to eukaryotes.

The authors might want to consider a few points:

While they used length of the trimmed sequence in the alignment as a possible discriminant, it might be better to directly measure intrinsic disorder or sequence entropy and use them as potential discriminants. This is of interest because in general eukaryotes have much great amount of low complexity sequence in their proteins than prokaryotes (especially low complexity sequence enriched in charge or polar residues or both). While other factors affect the amount of low complexity in eukaryotes does thermophily have a negative effect on it.

We have performed calculations of intrinsic protein disorder and included a new panel in Figure 3 showing the results. Using this measure (IUPred), Chaetomium ranks the highest. This is likely due to the high GC content of this species. Alvinella ranks average, indicating that intrinsic disorder is not a general discriminator of thermophilic and mesophilic eukaryotes.

Most homeothermic metazoans tend to maintain higher body temperatures than the rest. In their analysis of CvP Homo, a homeothermic metazoan is ranked next after the thermophilic species. However, a species with a much lower preferred temperature is also close. Is there any significance to this? Would it be possible to use some bird species in the comparison as they have much higher body temperatures (Gallus/Zebra Finch ~41 C). Would they show a higher rank in the CvP measure than other species in the current figure?

We thank the reviewer for this insightful comment. Indeed, among the homeothermic metazoans temperature differences can be quite large (36 to 41 C), and this could be reflected in their proteomes. Although a full analysis in homeothermic metazoans is beyond the scope of this paper, we determined the average CvP values of the proteomes of several homeothermic vertebrates and found a strong correlation between body temperature and the CvP values, as shown in Table 5. This question can be addressed in more detail in the future when more bird genomes and genomes from vertebrates with low body temperature (e.g. dolphins, whales) become available.

While the data is currently very limited, are there any possible explanations for the CvP being more successful as a discriminant than other measures in eukaryotes?

One possible explanation is that some of the other measures are over-fitted to a training dataset (e.g. the Tm-Index uses dipeptide composition). The CvP-bias is a simple measure with few parameters combining information from two classes of amino acids, the changes of which have previously been linked to thermoadaptation. CvP-bias also performs best on the prokaryotic datasets. A larger number of Alvinella and Chaetomium protein structures and their comparison to mesophilic ortologues will help to clarify the role of charged and polar residues in thermoadaptation.

Reviewer 3: Anthony M. Poole, School of Biological Sciences, University of Canterbury, Christchurch, New Zealand

Using transcriptomics data, this paper examines whether there is a signal of thermoadaptation in proteins from the thermotolerant annelid worm, Alvinella pompejana. As well as significantly increasing the available sequence data for this species, which is a worthy effort in itself, the authors examine whether there is a compositional signature for thermoadaptation in proteins from this species and from the thermotolerant yeast Chaetomium thermophilum. In contrast to a comparison of two prokaryote species (here there is a large literature on thermotolerant proteins), one a thermophile, and the other not, the authors find only a relatively faint indication of thermotolerance for these two eukaryotes. Of the nine thermostability measures presented, only one discriminates the two thermotolerant eukaryotes, whereas all were sufficient for discriminating between Thermus thermophilus (thermophilic) and Deinococcus radiodurans (dessication/radioresistant but not thermophilic).

This result is interesting in that it indicates that thermoadaptation of A. pompejana (and C. thermophilum) is not reflected in strong changes in the composition of individual proteins themselves. Of the nine measures employed here (Figures 2 &3), only one (CvP) suggests a signature of thermoadapation in the two thermotolerant eukaryote species. It is worth pointing out that the two bacterial cases examined show much greater differences in their optimal growth temperature, whereas, as discussed in the text, this may not be particularly high for A. pompejana.

In the case of the two eukaryotes, the degree to which proteins may be subject to high temperature is debated, particularly for A. pompejana. It is a pity that the authors did not include the thermotolerant red alga Cyanidioschyzon merolae, which was isolated from hot springs (45°C) for which there is also sequence data (Matsuzaki et al. 2004 Nature 428:653). Including this species would be fascinating from the point of view of the general question of understanding thermoadaptation at the protein level in eukaryotes, but is not likely to change the primary conclusions drawn about A. pompejana.

We thank the reviewer for this suggestion. We had a look at the Cyanidioschyzon merolae proteome, but could only identify 16 proteins that give reciprocal best blast hits to our orthologous set. Given that this red alga is phylogenetically very far from yeasts + metazoans, we could not include it in the comparisons. However, it would certainly be worth comparing Cyanidioschyzon merolae to mesophilic red algae in the future, once more sequence data will become available.

Given that the signal for proteins is so subtle, it would be interesting to look at non-coding RNAs - I suspect at least some will be present in the raw read data. This could be worthwhile in that GC-content of rRNA correlates with optimal growth temperature, whereas the GC content of genomic DNA, protein-coding genes or 3rd codon position of such genes does not (Hurst & Merchant 2001 Proc Roy Soc Lond B 268:493). If the signal in proteins is weak, as the present authors show, perhaps an analysis of RNA can help shed light on whether there is a molecular signature of thermoadaptation. This would be helpful in establishing whether the subtle difference observed here is an indicator that, of the various attributes that contribute to thermoadaptation, CvP is the most sensitive, and thus the first to show a signal under moderate thermoadaptation.

Following the reviewer’s suggestion we have looked at 28S rRNA and obtained GC-content from the trimmed alignment (1200 nucleotides) for six species: D. melanogaster, 41.80; S. cerevisiae, 46.39; C. elegans, 47.42; C. thermophilum, 49.24; A. pompejana, 52.89; H. sapiens, 60.63. Since we do not see a trend and Alvinella and Chaetomium rank average, we did not include this analysis in the paper.

## Competing interests

The authors have no competing interests.

## Authors’ contributions

TH performed bioinformatics and thermostability calculations, CB performed thermal shift assays, JE purified proteins, NR extracted *A. pompejana* mRNA, DJ collected the *A. pompejana* specimens and wrote the paper, EC designed the project, GJ performed bioinformatics, gene annotation and assembly, supervised the project and wrote the paper, FB designed and supervised the project and wrote the paper. All authors read and approved the final manuscript.

## Supplementary Material

Additional file 1 **Transcriptome dataset 1 MPI transcriptome dataset generated by the clustering of all new *A. pompejana *sequence data in this study.** The file was compressed with tar -jcvf archive_name.tar.bz2 file_to_compress, use tar –jxvf to uncompress it. Contains 74762 sequences. Click here for file

Additional file 2 **Transcriptome dataset 2 JGI + Genoscope transcriptome dataset generated by the clustering of all publicly available *A. pompejana *ESTs (as of Feb 2011).** The file was compressed with tar -jcvf archive_name.tar.bz2 file_to_compress, use tar –jxvf to uncompress it. Contains 39155 sequences. Click here for file

Additional file 3 **Transcriptome dataset 3 MPI + JGI + Genoscope transcriptome dataset generated by the clustering of all publicly available *A. pompejana *ESTs (as of Feb 2011) and all new *A. pompejana *sequence data in this study.** The file was split into two and compressed with tar -jcvf archive_name.tar.bz2 file_to_compress, use tar –jxvf to uncompress it. Contains 97285 sequences. Click here for file

Additional file 4 **Predicted protein dataset 1 MPI predicted protein dataset generated by ORF prediction on all new clustered *A. pompejana *sequence data in this study.** Potential full-length protein sequences are indicated as ‘closed = (1/1)’. The file was compressed with tar -jcvf archive_name.tar.bz2 file_to_compress, use tar –jxvf to uncompress it. Contains 29013 sequences. Click here for file

Additional file 5 **Predicted protein dataset 2 JGI + Genoscope predicted protein dataset generated by ORF prediction on the clustered transcripts derived from all publicly available *A. pompejana *ESTs (as of Feb 2011).** Potential full-length protein sequences are indicated as ‘closed (1/1)’. The file was compressed with tar -jcvf archive_name.tar.bz2 file_to_compress, use tar –jxvf to uncompress it. Contains 36328 sequences. Click here for file

Additional file 6 **Predicted protein dataset 3 MPI + JGI + Genoscope predicted protein dataset generated by ORF prediction on the clustered transcripts derived from all publicly available *A. pompejana *ESTs (as of April 2011) and all new *A. pompejana *sequence data in this study.** Potential full-length protein sequences are indicated as ‘closed = (1/1)’. The file was compressed with tar -jcvf archive_name.tar.bz2 file_to_compress, use tar –jxvf to uncompress it. Contains 42665 sequences. Click here for file

Additional file 7 **Phylogenetic tree of the 10 species used in the orthologous set.** The tree is based on the recently published phylogeny of annelids [36] and the consensus animal phylogeny [37]. Click here for file

Additional file 8 **Annotated predicted *A. pompejana * proteins 42665 predicted *A. pompejana *protein sequences, defined by their first BLASTP hit in the SwissProt database and annotated with the first BLASTP hits in the *Capitella teleta*, *Homo sapiens *, *Danio rerio *and *Drosophila melanogaster *proteomes.** The file was compressed with tar -jcvf archive_name.tar.bz2 file_to_compress, use tar –jxvf to uncompress it. Contains 42665 sequences. Click here for file

Additional file 9 **Orthologous set.** Trimmed multiple sequence alignments of the orthologous set of 457 proteins from the 10 species used in the study. Click here for file

Additional file 10 **Frequency of individual amino acids as a function of GC-content.** The frequency of amino acids is plotted as a function of GC3-content (GC-content at the third codon position) in the species of the orthologous set. The regression lines with 90% confidence intervals are shown. Click here for file
