# Supplementary figures and images for: Deep transcriptome-sequencing and proteome analysis of the hydrothermal vent annelid Alvinella pompejana identifies the CvP-bias as a robust measure of eukaryotic thermostability
Source: Biol Direct. 2013 Jan 16;8:2. doi: 10.1186/1745-6150-8-2 (PMC3564776; doi:10.1186/1745-6150-8-2)

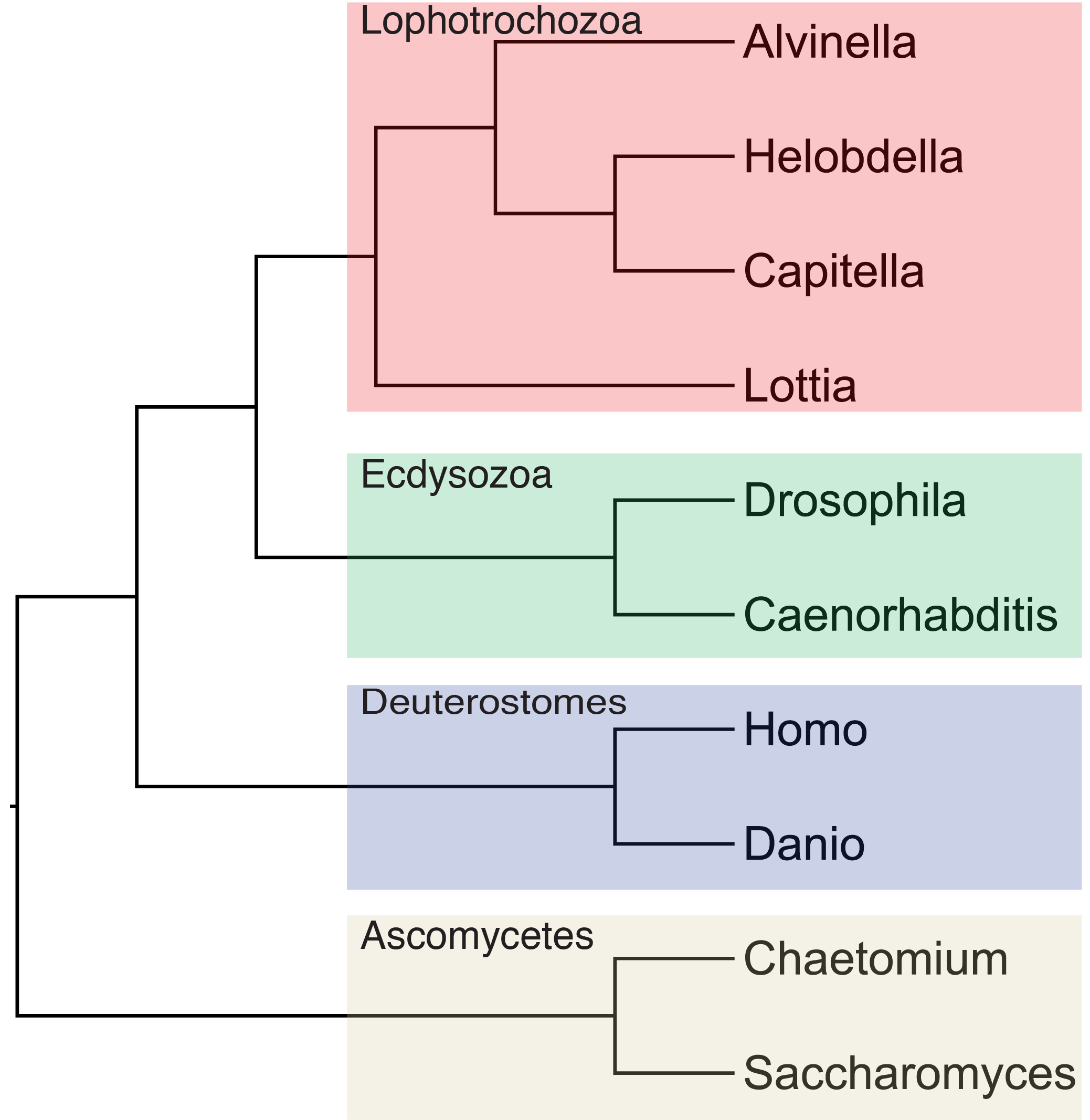

Supplement: Additional file 8 — Annotated predicted A. pompejana proteins 42665 predicted A. pompejana protein sequences, defined by their first BLASTP hit in the SwissProt database and annotated with the first BLASTP hits in the Capitella teleta, Homo sapiens , Danio rerio and Drosophila melanogaster proteomes. The file was compressed with tar -jcvf archive_name.tar.bz2 file_to_compress, use tar –jxvf to uncompress it. Contains 42665 sequences. [file 1745-6150-8-2-S8.png]
